# Use of syngeneic cells expressing membrane-bound GM-CSF as an adjuvant to induce antibodies against native multi-pass transmembrane protein

**DOI:** 10.1038/s41598-019-45160-9

**Published:** 2019-07-09

**Authors:** Chien-Chiao Huang, Kai-Wen Cheng, Yuan-Chin Hsieh, Wen-Wei Lin, Chiu-Min Cheng, Shyng-Shiou F. Yuan, I-Ju Chen, Yi-An Cheng, Yun-Chi Lu, Bo-Cheng Huang, Yi-Ching Tung, Tian-Lu Cheng

**Affiliations:** 1 Drug Development and Value Creation Research Center, Kaohsiung Medical University, Kaohsiung, Taiwan; 2 Graduate Institute of Medicine, College of Medicine, Kaohsiung Medical University, Kaohsiung, Taiwan; 3 Department of Laboratory Medicine, School of Medicine, College of Medicine, Kaohsiung Medical University, Kaohsiung, Taiwan; 4 Department of Medical Research, Kaohsiung Medical University Hospital, Kaohsiung, Taiwan; 5 Department and Graduate Institute of Aquaculture, National Kaohsiung University of Science and Technology, Kaohsiung, Taiwan; 6 Translational Research Center, Kaohsiung Medical University Hospital, Kaohsiung, Taiwan; 7 Center for Cancer Research, Kaohsiung Medical University, Kaohsiung, Taiwan; 8 Institute of Biomedical Sciences, National Sun Yat-sen University, Kaohsiung, Taiwan; 9 Department of Public Health and Environmental Medicine, College of Medicine, Kaohsiung Medical University, Kaohsiung, Taiwan; 10 Department of Biomedical Science and Environmental Biology, Kaohsiung Medical University, Kaohsiung, Taiwan; 11 Center for Molecular Medicine, China Medical University, Taichung, Taiwan

**Keywords:** Protein design, Antibody therapy

## Abstract

Membrane antigens (mAgs) are important targets for the development of antibody (Ab) drugs. However, native mAgs are not easily prepared, causing difficulties in acquiring functional Abs. In this study, we present a platform in which human mAgs were expressed in native form on cell adjuvants made with membrane-bound cytokines that were then used immunize syngeneic mice directly. The membrane-bound cytokines were used as immune stimulators to enhance specific Ab responses against the desired mAgs. Then, mAgs-expressing xenogeneic cells were used for Ab characterization to reduce non-specific binding. We established cell adjuvants by expressing membrane-bound cytokines (mIL-2, mIL-18, or mGM-CSF) on BALB/3T3 cells, which were effective in stimulating splenocyte proliferation *in vitro*. We then transiently expressed ecotropic viral integration site 2B (EVI2B) on the adjuvants and used them to directly immunize BALB/c mice. We found that 3T3/mGM-CSF cells stimulated higher specific anti-EVI2B Ab response in the immunized mice than the other cell adjuvants. A G-protein coupled receptor (GPCR), CXCR2, was then transiently expressed on 3T3/mGM-CSF cell adjuvant to immunize mice. The immune serum exhibited relatively higher binding to xenogeneic 293 A/CXCR2 cells than 293 A cells (~3.5-fold). Several hybridoma clones also exhibited selective binding to 293 A/CXCR2 cells. Therefore, the cell adjuvant could preserve the native conformation of mAgs and exhibit anti-mAg Ab stimulatory ability, providing a more convenient and effective method to generate functional Abs, thus possibly accelerating Ab drug development.

## Introduction

Membrane antigens (mAgs), such as G protein-coupled receptors (GPCRs) that constitute a large family of protein receptors are attractive and important targets for antibody (Ab) therapy in various diseases^1,2^. Up to 50–60% of the current drugs on the market are GPCR-targeted, but only two are Ab drugs^3^. The major impediment in generating functional Abs for Ab drug development may be the difficulty in obtaining native forms of mAgs to immunize animals and follow the Ab characterization process. In fact, GPCRs are characterized by a complicated structure with 7 transmembrane domains that are difficult to express and purify in native form using traditional methods. As a result, mAgs are expressed on the surface of carrier cells to preserve the native conformation of mAgs, which are directly used for immunization. For example, Dreyer and colleagues expressed a predicted GPI-anchored protein of the malaria parasite *P. falciparum* on human embryonic kidney (HEK) cells to immunize xenogeneic NMRI mice. They observed non-specific Ab responses against HEK cells, accounting for 21–34% of the tested wells^4^. Spiller and colleagues expressed GPI-anchored rat CD55 on NIH SWISS-mouse-derived NIH-3T3 fibroblast cells to immunize allogeneic BALB/c mice. Although BALB/c mice were expected to be more tolerant against allogeneic NIH-3T3 cells, they still generated anti-alloantigen Ab responses to the NIH-3T3 cells^5^. Both xenogeneic and allogenic carrier cells also induced non-specific Ab responses in the immunized mice, thus using the same cells for Ab characterization may increase the difficulty in screening for specific and functional anti-mAg Abs.

**Figure 1:**
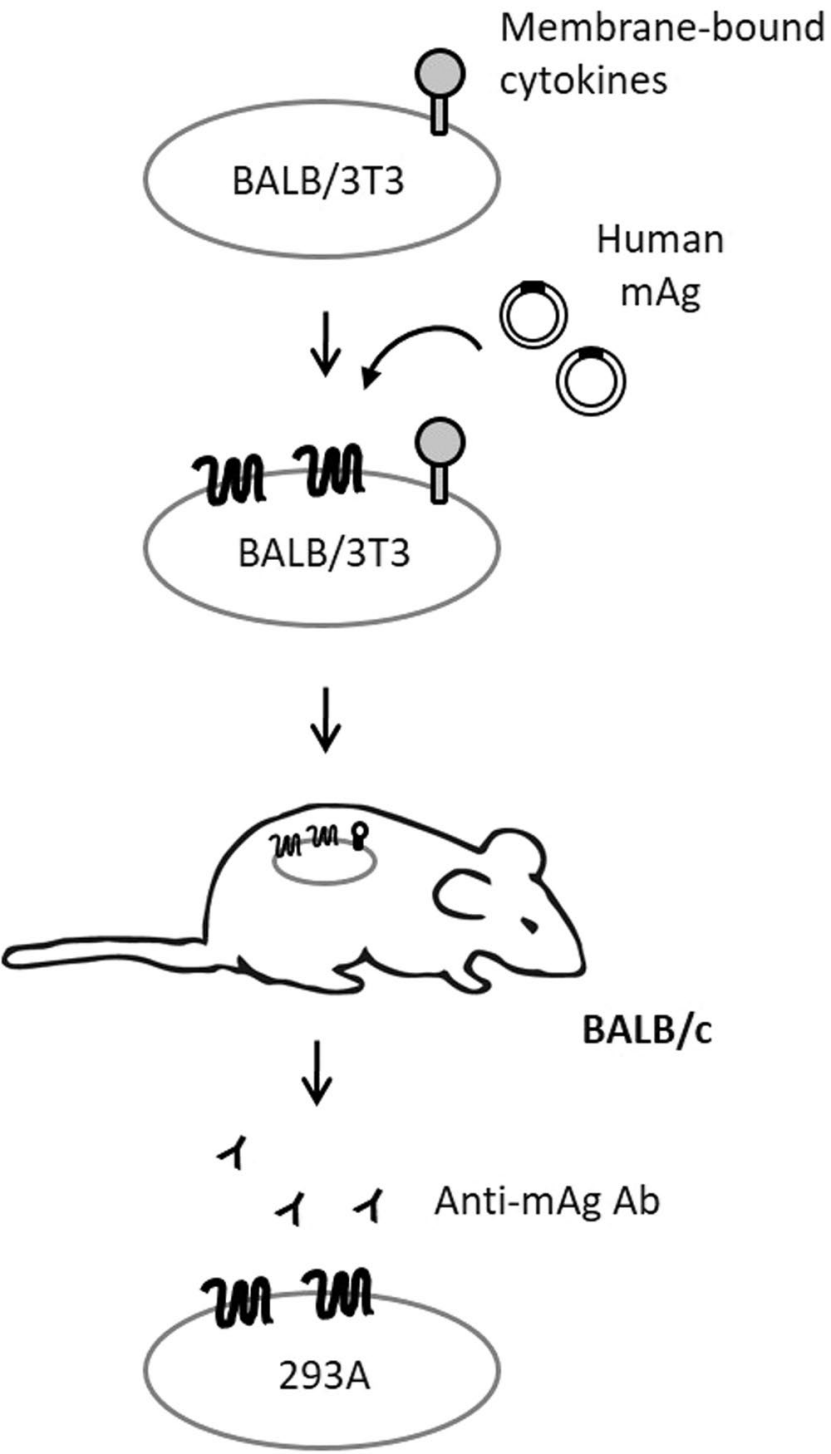
Platform for the generation of anti-membrane antigens Ab. Cytokines (IL-2, IL-18, and GM-CSF) were anchored on the plasma membrane of BALB/3T3 cells as immune stimulators to be cell adjuvants. The mAg (EVI2B or CXCR2) encoding plasmids were then delivered into the cell adjuvants by transient transfection to express the desired mAg in native form on the cell surface, which are used directly to immunize syngeneic BALB/c mice to induce anti-mAg Abs. Xenogeneic 293 A cells stably expressing mAgs were used for screening and Ab characterization to eliminate non-specific binding.

Syngeneic cells that contain the same genomic background and low immunogenicity to the host animals may help reduce non-specific Ab response in the immunized animals. However, using syngeneic cells alone as a carrier to immunize animals may induce weak immune responses which are inefficient to elicit Ab responses^6^. An efficient stimulator, therefore, is necessary to enhance Ab responses against the desired mAgs expressed on the syngeneic carrier cells. Williams and colleagues reported that cancer patients who received tumors admixed with IL-2 had an average 33% higher serum titer against autologous tumor cells whereas only an 8% increase was seen in the non-IL-2 injected patients^7^. Hoshino and *et al*. showed that administration of IL-18 enhanced IgE, IgG1, IgG2a, and IgM serum concentration by 1.2–8 fold in B6 mice compared to mice treated with PBS alone^8^. Kyung and *et al*. reported that ovalbumin (OVA) mixed with granulocyte-macrophage colony-stimulating factor (GM-CSF) in an adjuvant produced 1000-fold higher OVA specific IgG titer in mice serum compared with the adjuvant without GM-CSF^9^. Collectively, IL-2, IL-18, and GM-CSF are ideal immune stimulators in adjuvants that enhance Ab responses. Therefore, our strategy was to establish a syngeneic cell adjuvant by expressing membrane-bound cytokines on BALB/3T3 cells (Fig. 1). Human mAg encoding plasmids were delivered into the cell adjuvant by transient transfection to express the desired mAgs on the cell surface. We expected this design could retain the native conformation of mAgs and also increase the immune response to the desired mAgs when they were injected into syngeneic BALB/c mice. Then, using xenogeneic cells, such as 293 A cells, a subclone of HEK 293 cell line, to express mAgs for Ab characterization was able to reduce non-specific binding, thus increasing the possibility to screen for functional Abs.

In this study, we fused murine cytokines (IL-2, IL-18, or GM-CSF) with the transmembrane domain of murine B7-1 to anchor each cytokine on BALB/3T3 cells to establish cell adjuvants. We then examined the surface expression and function of these membrane-bound cytokines on BALB/3T3 cells. After that, we applied these cell adjuvants to two potential targets, a type I single-passed transmembrane protein, ecotropic viral integration site 2B (EVI2B) and a type IV multi-pass transmembrane GPCR, C-X-C chemokine receptor type 2 (CXCR2). Antibody drugs for these two mAgs are not yet available on the market. First, we transiently expressed EVI2B on each adjuvant, and used them directly to immunize BALB/c mice. The potential cell adjuvant that was relatively effective in stimulating specific Ab response was then used to express CXCR2 for mouse immunization. Finally, the anti-CXCR2 Ab response from hybridoma supernatants was characterized by CXCR2-expressing 293 A cells. Collectively, our results show that this syngeneic cell adjuvant platform which preserves the native structure of mAgs may provide a new strategy to immunize animals and generate functional Abs.

## Materials and Methods

### Cells

BALB/3T3 mouse fibroblast cells, GP2-293 retroviral packaging cells, 293 T and 293 A human embryonic kidney epithelial cells, 293 A and FO myeloma cells were cultured in DMEM (Sigma-Aldrich) containing 10% heat-inactivated bovine calf serum (GE Healthcare Life Science), and 100 units/mL penicillin, and streptomycin (Thermo Fisher Scientific) at 37 °C in a humidified atmosphere containing 5% CO_2_.

### Generation of membrane-bound cytokines-expressing BALB/3T3 cells

The DNA sequences of murine IL-2, IL-18, or GM-CSF was fused to the B7 transmembrane domain and then subcloned into the retroviral vector pLNCX (BD Biosciences). Recombinant retroviral particles were produced after co-transfection of pLNCX construct with pVSVG into GP2-293 cells. After 48 hours, the collected culture medium was filtered through a 0.22-μm syringe filter, mixed with 8 μg/mL polybrene, and then added to BALB/3T3 cells for virus infection. The cells were selected in G418 and sorted by FACScaliber flow cytometer to generate 3T3/mIL-2, 3T3/mIL-18, 3T3/mGM-CSF, or 3T3 mock control cells.

### Determination of membrane-bound cytokine expression

Membrane cytokine-expressing BALB/3T3 or mock control cells (5 × 10^6^) were harvested and boiled in reducing SDS buffer, electrophoresed on SDS-PAGE, and transferred to nitrocellulose (NC) membranes (Millipore). Membranes were blocked in 5% skin milk (BD Biosciences) in phosphate buffered saline (PBS), and sequentially stained with mouse anti-HA tag Ab, followed by goat anti-mouse IgG Fc-HRP (Jackson Immuno-Research Laboratories). Bands were visualized by ECL (Millipore) and monitored using UVP BioImaging system. Surface expression of membrane-bound cytokines on BALB/3T3 cells were determined by staining the cells with mouse anti-IL-2 Ab (Arigo Biolaboratories, ARG21459), anti-IL-18 Ab (Arigo Biolaboratories, AGR55332), or anti-GM-CSF Ab (Arigo Biolaboratories, ARG21498) in staining buffer (PBS containing 0.1% bovine serum albumin), followed by FITC-conjugated goat anti-rat IgG (H + L) Ab (Jackson Immuno-Research Laboratories), FITC-conjugated goat anti-rabbit IgG (H + L) Ab (Jackson Immuno-Research Laboratories), respectively, The surface florescence intensity of viable cells was measured by a FACScaliber flow cytometer.

### *In vitro* functional analysis of membrane-bound cytokines

BALB/c mice were sacrificed using CO_2_ and the spleens were harvested. Spleens were mashed and filtered through a cell strainer, and treated with ACK lysis buffer to remove red blood cells. Splenocytes were suspended in RPMI 1640 growth medium containing mitogen concanavalin A (Sigma-Aldrich) at a concentration of 5 μg/mL, and splenocytes (6 × 10^5^ per well) were seeded in 96-well plates containing 200 µl medium per well. 3T3/mIL-2, 3T3/mIL-18, 3T3/mGM-CSF, or 3T3 mock control cells were suspended in PBS and continuously freeze-thawed 2 times to stop cell proliferation. These non-proliferating cells (6 × 10^4^ per well) were co-incubated with splenocytes for 24, 48, 72, 96, and 108 h. ATPlite luminescence assay (PerkinElmer) was performed to evaluate splenocyte proliferation at the indicated time point according to the manufacturer’s instructions.

### Construction of EVI2B- or CXCR2-expressing cell adjuvants

The sequence of human EVI2B or CXCR2 was cloned into vector pcDNA3.1(-) for protein expression. A sequence coding for His-tag was inserted before the EVI2B or CXCR2 sequence to establish pcDNA3.1/EVI2B and pcDNA3.1/CXCR2, respectively. Cell adjuvants (3T3/mIL-2, 3T3/mIL-18, 3T3/mGM-CSF, and 3T3 mock control), were transfected with pcDNA3.1/EVI2B or pcDNA3.1/CXCR2 plasmids by using Lipofectamine 2000 reagent (Thermo Fisher Scientific) according to the manufacturer’s instructions. After 48 h, surface expression of EVI2B or CXCR2 was determined by Western blotting and by flow cytometry with mouse anti-EVI2B Ab (Thermo Fisher Scientific, MEM-216), anti-CXCR2 Ab (R&D Systems, MAB331), or anti-His tag Ab as described above.

### Animal experiments

BALB/c mice (4 to 5 weeks old) were purchased from the National Laboratory Animal Center, Taipei, Taiwan. All animal experiments were carried out in accordance with institutional guidelines and approved by the Animal Care and Use Committee of Kaohsiung Medical University. Cells were washed and resuspended in PBS. BALB/c mice were s.c. injected on the back with 10^6^ cells in 100 μL once every 7 days a total of 4 times. Two days after the fourth injection, tail vein blood was collected and the serum was frozen for storage at −80 °C.

### Generation of EVI2B- or CXCR2-expressing 293A cells

293 A/EVI2B and 293 A/CXCR2 cells that stably express EVI2B or CXCR2 on the surface were generated by lentivirus transduction. The sequence of EVI2B or CXCR2 with His-tag was cloned into vector pAS3w.Ppuro (National RNAi Core Facility, Academia, Sinica, Taiwan) to establish pAS3w.Ppuro/EVI2B and pAS3w.Ppuro/CXCR2, respectively. Recombinant lentiviral particles were packaged by co-transfection of pAS3w.Ppuro construct with pCMVΔR8.91 and pMD.G in 293 T cells. After 48 hours, the collected culture medium was filtered through a 0.22-μm syringe filter, mixed with 8 μg/mL polybrene, and then added to 293 A cells for virus infection. The cells were selected in puromycin to generate stable cell lines.

### Anti-EVI2B or anti-CXCR2 Ab determination in immunized mice serum

Ab response in serum of the immunized mice was determined by cell-based ELISA or flow cytometry. Cells (2 × 10^5^ per well) were seeded in 96-well plates in DMEM culture medium and incubated overnight. The plate was incubated with 1% paraformaldehyde for 3 minutes at RT and blocked with 5% of skim milk in PBS. Mouse serum diluted in 2% skim milk in PBS was added to the wells. The plates were stained with HRP-conjugated goat anti-mouse IgG. ABTS solution (0.4 g/mL, 2,2′-azino-di(3-ethylbenzthiazoline-6-sulfonic acid) (Sigma-Aldrich), 0.003% H_2_O_2_, and 100 mM phosphate-citrate, pH = 4.0) was added, incubated, and measured for the OD 405 nm value by microplate reader. Similarly, anti-CXCR2 Ab titer in serum was also analyzed by flow cytometry as described above.

### Fusion and anti-CXCR2 Ab characterization in hybridoma supernatants

A mouse with serum that had higher binding to 293 A/CXCR2 cells was selected for fusion. Spleen cells were harvested by trituration under sterile conditions and were mashed. Spleenocytes were fused with FO myeloma cells using poly-ethylene glycol 1500 (Roche) at 37 °C. The fusion mix was plated into 96-well plates and cultured in HAT medium (Sigma-Aldrich) to generate hybridomas. After 2 weeks, supernatants from each well were collected and the anti-CXCR2 Ab response was determined by cell-based ELISA as described above.

### Statistical analysis

Graphpad Prism V5 software was used to perform statistical analysis. All data were analyzed for significance by using One-way ANOVA followed by Student’s Newman-Keuls test. A P value of less than 0.05 was considered statistically significant.

## Results

### Establishment of membrane-bound cytokines on BALB/3T3 cells as adjuvants

**Figure 2:**
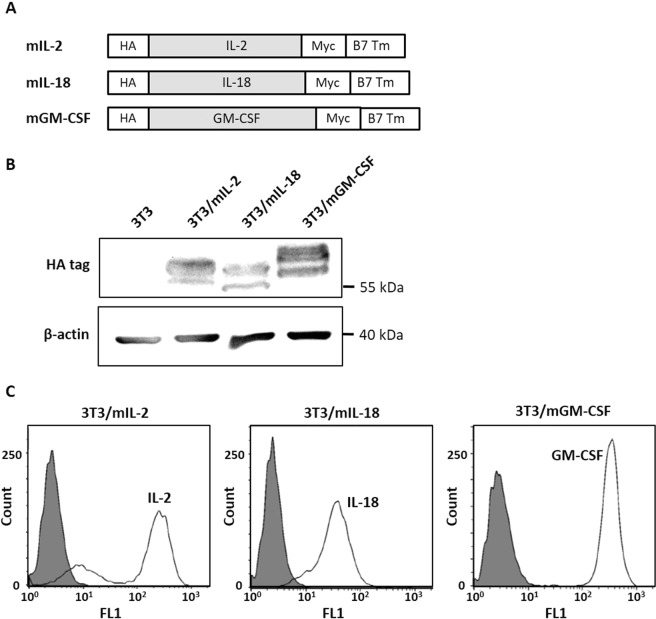
Establishment of membrane-bound cytokines on BALB/3T3 as cell adjuvants. (**A**) Schematic representation of membrane-bound cytokines constructs, which code for an HA epitope (HA), the murine cytokines (IL-2, IL-18, or GM-CSF), a myc epitope (Myc), and the transmembrane domain of murine B7-1 (B7 Tm). (**B**) Expression of membrane-bound cytokines in the transduced BALB/3T3. mIL-2, mIL-18, and mGM-CSF were detected by western blotting using anti-HA tag Ab. β-actin was used as the internal control of each cell lysate. Full blots are shown in Supplementary Fig. 1. (**C**) Surface expression of membrane-bound cytokines on the transduced BALB/3T3. mIL-2, mIL-18, and mGM-CSF were detected by flow cytometry after staining the cells with anti-IL-2 Ab, anti-IL-18 Ab, and anti-GM-CSF Ab, respectively.

To express membrane-bound IL-2, IL-18, and GM-CSF (mIL-2, mIL-18, and mGM-CSF) on BALB/3T3 cells, murine IL-2, IL-18, and GM-CSF genes were subcloned into the N-terminal of membrane anchoring B7 transmembrane domain (B7 Tm) in the retroviral vector pLNCX (Fig. 2A). BALB/3T3 cells were infected with each of the recombinant retroviral particles and selected in G418 to obtain 3T3/mIL-2, 3T3/mIL-18, 3T3/mGM-CSF, and 3T3 mock control cells. The expression of cytokine fusion proteins was detected by immunoblotting for the presence of the HA-epitope in whole cell lysates as shown in Fig. 2B. We observed that the multiple protein bands were enriched in the three cytokine proteins, due to post-translational modification of IL-2^10^, GM-CSF^11^, and B7-1^12^.

We next isolated the cells with high levels of membrane-bound cytokines by fluorescence-activated cell sorting. The surface expression of each cytokine on BALB/3T3 cells was detected by flow cytometry using anti-IL-2 Ab, anti-IL-18 Ab, and anti-GM-CSF Ab, respectively, as shown in Fig. 2C. Stained 3T3/mIL-2, 3T3/mIL-18, and 3T3/mGM-CSF cells exhibited relatively strong fluorescence intensity compared to non-stained cells, indicating the expression of membrane-bound cytokines on the cells. Therefore, we successfully expressed cytokines, IL-2, IL-18, and GM-CSF on BALB/3T3 cells.

### Bioactivity of membrane-bound cytokines on BALB/3T3 cells

**Figure 3:**
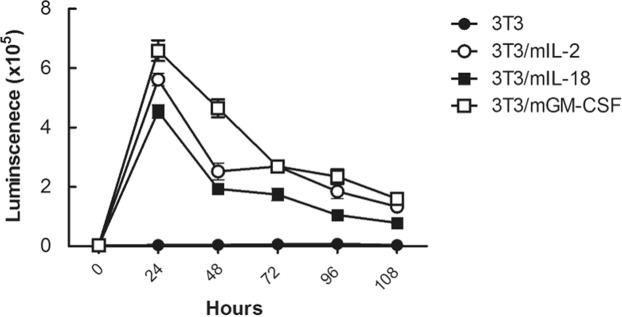
Bioactivity of cell adjuvants. The effect of the membrane-bound cytokines on splenocyte proliferation were determined. Splenocytes were harvested from BALB/c mice, and were co-incubated with non-proliferating cell adjuvants (3T3/mIL-2, 3T3/mIL-18, or 3T3/mGM-CSF), or 3T3 mock control for 24, 48, 72, 96 and 108 h. Splenocyte numbers were measured by using ATPlite luminescence assay. Data are shown as mean ± SD. n = 3 biological replicates.

To determine the bioactivity of membrane-bound cytokines, we examined whether 3T3/mIL-2, 3T3/mIL-18, and 3T3/mGM-CSF cells could stimulate immune cell responses. We performed mouse splenocyte proliferation assay that reflects the initiation of immune response *in vivo*^13^. 3T3/mIL-2, 3T3/mIL-18, and 3T3/mGM-CSF, or 3T3 mock control cells were continuously freeze-thawed to stop cell proliferation. Splenocytes were harvested from healthy BALB/c mice and were co-incubated with each non-proliferating cell line for various periods of time. The splenocyte proliferation was examined by ATPlite luminescence assay as shown in Fig. 3. The three cell lines with membrane-bound cytokines exhibited higher luminescence compared to the 3T3 mock control, indicating that each cytokine was functionally active to induce splenocyte proliferation. Moreover, the level of splenocyte proliferation peaked at 24 h and continued up to 108 h in the groups with membrane-bound cytokines. Collectively, these results showed that high levels of functional cytokines, IL-2, IL-18, and GM-CSF, can be expressed on BALB/3T3 cells.

### Determination of anti-EVI2B Ab production efficacy

**Figure 4:**
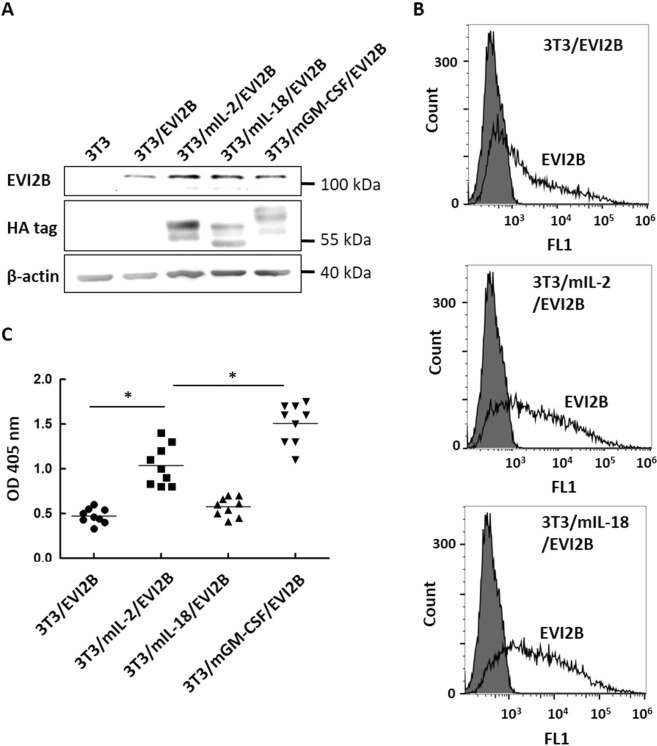
Characterization of anti-EVI2B Ab responses in immune serum. (**A**) Expression of EVI2B in the transient transfected cell adjuvants. EVI2B and each cytokine were detected by western blotting using anti-EVI2B Ab and anti-HA tag Ab, respectively. β-actin was used as the internal control of each cell lysate. Full blots are shown in Supplementary Fig. 2. (**B**) Surface expression of EVI2B in the transient transfected cell adjuvants. EVI2B was detected by flow cytometry after staining the cells with anti-EVI2B Ab. (**C**) BALB/c mice were s.c. injected with EVI2B-expressing cell adjuvants **(**3T3/EVI2B, 3T3/mIL-2/EVI2B, 3T3/IL-18/EVI2B, and 3T3/mGM-CSF/EVI2B). Anti-EVI2B Ab responses in mouse serum were measured by cell-based ELISA using 293 A/EVI2B cells (n = 9). Immune serum was 1000-fold diluted. P values were determined by Student’s Newman-Keuls test: *, *P* < 0.05.

We then chose EVI2B, a biomarker correlated with postoperative relapse of colorectal cancers^14^, as our first candidate mAg. EVI2B was transiently expressed on cell adjuvants or 3T3 mock control to establish 3T3/mIL-2/EVI2B, 3T3/IL-18/EVI2B, 3T3/mGM-CSF/EVI2B, and 3T3/EVI2B cells, respectively. The protein expression of EVI2B and each cytokine were examined by immunoblotting whole cell lysates as shown in Fig. 4A. A similar level of EVI2B protein was detected in the four cell adjuvants. The surface expression level of EVI2B was assessed by flow cytometry using anti-EVI2B Ab. As shown in Fig. 4B, a relatively strong fluorescence intensity was detected in the transduced cells, indicating the successful expression of EVI2B on the surface of these cell adjuvants.

To evaluate the Ab stimulatory efficacy of these cell adjuvants, the four cell adjuvants with transient expression of EVI2B were used to immunize BALB/c mice. Mice were immunized every 7 days, 4 times in total. Mouse serum was collected and anti-EVI2B Ab levels were determined via cell-based ELISA using 293 A/EVI2B cells. The anti-EVI2B Ab production is represented as OD405 value as shown in Fig. 4C. The OD405 value was significantly increased in the 3T3/mIL-2/EVI2B group and the 3T3/mGM-CSF/EVI2B group compared to the 3T3/EVI2B group by 2-fold and 3-fold, respectively, indicating the effective Ab stimulatory potency of the two cell adjuvants. Importantly, the Ab tilter in the 3T3/mGM-CSF/EVI2B group was significantly higher than that in the other groups, approximately 1.5-fold and 3-fold higher than the 3T3/mIL-2/EVI2B group and 3T3/mIL-18/EVI2B group, respectively. These results show that the cell adjuvant with mGM-CSF stimulated anti-EVI2B Ab responses most potently. Therefore, 3T3/mGM-CSF could serve as the ideal cell adjuvant for Ab production.

### Multi-pass transmembrane protein Ab induction by cell adjuvants

**Figure 5:**
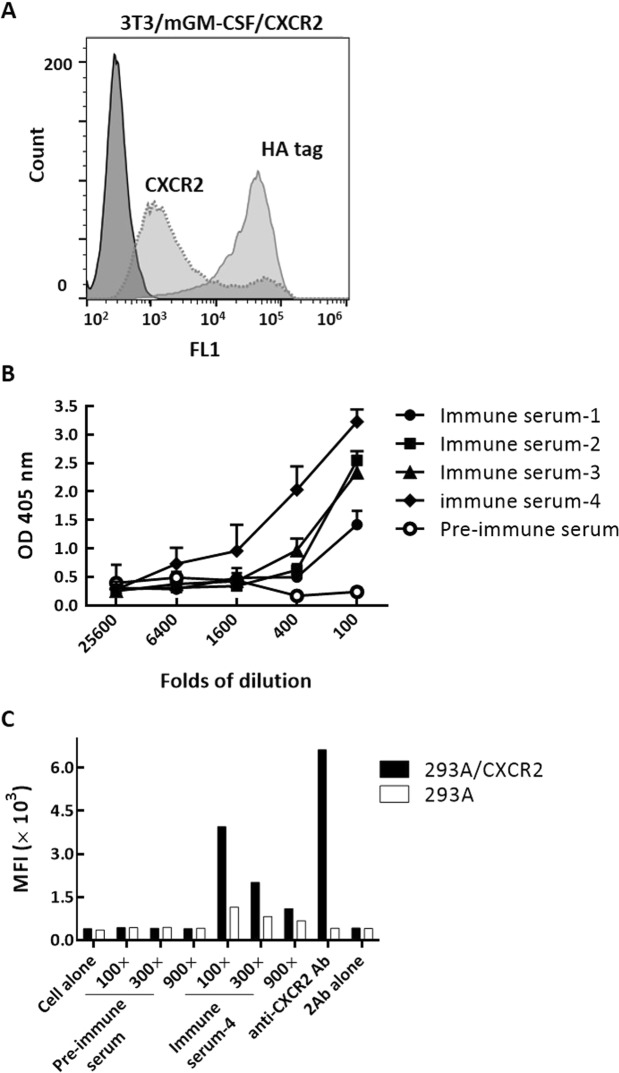
Anti-CXCR2 Ab responses in mice immunized with 3T3/mGM-CFS/CXCR2 cells. (**A**) Surface expression of CXCR2 and mGM-CSF on the 3T3/mGM-CSF/CXCR2 cells was determined by a commercial anti-CXCR2 Ab and anti-HA tag Ab via flow cytometry. (**B**,**C**) Anti-CXCR2 Ab responses in mouse serum. Four BALB/c mice were s.c. injected with 3T3/mGM-CSF/CXCR2 cells. Mouse serum were collected from BALB/c mice before (pre-immunization serum) and after immunization (immune serum). (**B**) Anti-CXCR2 Ab responses were determined by cell-based ELISA using 293 A/CXCR2 cells. Immune serum was 100- to 25600-fold diluted. Data are shown as mean ± SD. n = 3 biological replicates. (**C**) anti-CXCR2 Ab responses in mouse serum (immune serum-4) were determined by flow cytometry after staining the 293 A/CXCR2 or 293 A cells with mouse serum (100- to 900-fold dilution). Results represent the surface mean fluorescence intensities (MFI).

We chose CXCR2 to determine whether our cell adjuvant could be applied in multi-pass membrane proteins. CXCR2, a GPCR with 7 transmembrane domains, was identified as a key factor in tumor growth, angiogenesis, and metastasis^2^, making it an attractive target for Ab drug development^15^. We then transiently expressed CXCR2 on the cell surface of 3T3/mGM-CSF to generate 3T3/mGM-CSF/CXCR2 cells. As shown in Fig. 5A, surface expression of CXCR2 and mGM-CSF on the cells was determined by a commercial anti-CXCR2 Ab and anti-HA tag Ab via flow cytometry. We then immunized mice with 3T3/mGM-CSF/CXCR2 every 7 days, 4 times in total. Mouse serum was collected before and after immunization to examine the generation of the anti-CXCR2 Ab response. We further examined the anti-CXCR2 Ab response in immune serum using 293 A/CXCR2 cells to eliminate non-specific binding signal influence. Figure 5B shows the binding intensity of mice serum to 293 A/CXCR2 cells determined by cell-based ELISA. The immune serum displayed relatively high-level binding compared to the pre-immunization serum. Moreover, we also compared the serum binding to 293 A/CXCR2 cells using the immune serum-4, which had a stronger titer than the others, by flow cytometry. Non-transfected 293 A cells were also used as negative control cells. The surface fluorescence of each group is summarized in Fig. 5C. The surface fluorescence was ~ 3.5 fold higher in immune serum against 293 A/CXCR2 cells compared to 293 A control cells in the 100 × diluted serum, indicating that anti-CXCR2 Abs were generated in the immunized mice.

**Figure 6:**
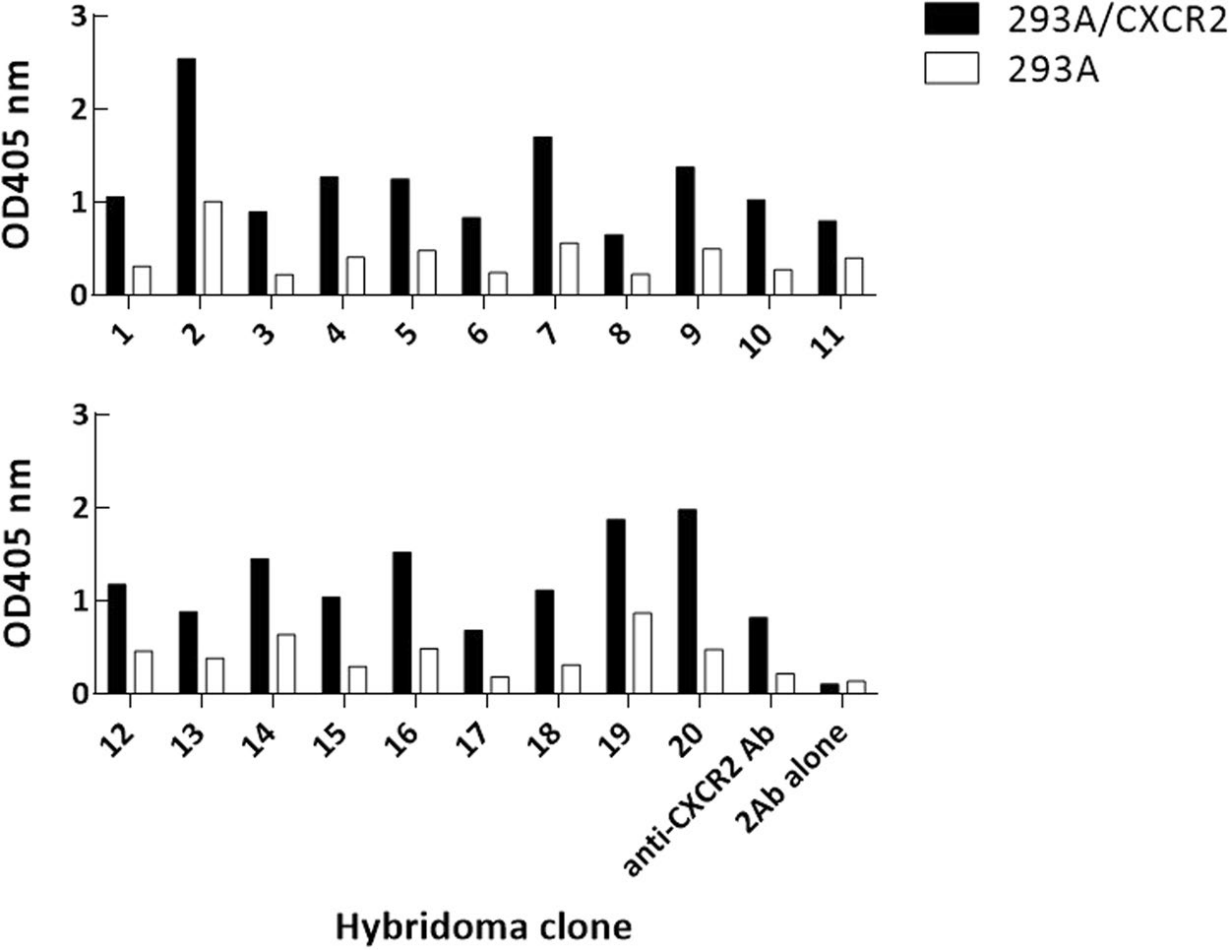
Specificity of hybridoma culture supernatants against CXCR2. Mouse spleen was fused with FO myeloma cells to generate hybridomas, which were then distributed in 15 96-well plates. Anti-CXCR2 Ab response in culture supernatants was determined by cell-based ELISA using 293 A/CXCR. Non-transfected 293 A cells were used as negative control cells. Anti-CXCR2 Ab was used as a positive control.

We further fused spleen from mouse 4 with FO myeloma cells to generate hybridomas. The fused mix was distributed in 1440 wells in 15 96-well plates. Then, we determined the anti-CXCR2 Ab response from each well by cell-based ELISA using 293 A/CXCR. Non-transfected 293 A cells were used as a negative control. Among the tested wells, 19.7% of all tested wells contained Abs binding to 293 A/CXCR2 cells (OD405 value > 0.5), whereas only ~ 9.0% of all tested wells contained Abs reacted with 293 A cells (OD405 value > 0.5). Moreover, we found that ~ 10.6% of all tested wells contained Abs that were more reactive to 293 A/CXCR2 cells than 293 A cells (the OD405 value ratio of 293 A/CXCR2 to 293 A > 2). Figure 6 shows the binding intensities of supernatants from 20 culture supernatants of tested wells determined by cell-based ELISA, which exhibited stronger binding to 293 A/CXCR2 cells compared to 293 A cells. Collectively, the data show the success of anti-CXCR2 Ab induction in the immunized mice, suggesting that this cell adjuvant platform is applicable for multi-pass membrane protein.

## Discussion

In this study, we developed 3T3/mGM-CSF as a cell adjuvant to express native form mAgs on the cell surface to use in immunization. The cell adjuvant was designed to utilize syngeneic BALB/3T3 cells to immunize BALB/c mice that can enhance immune response of the desired human mAgs and reduce unnecessary immune response against the allo-antigen on carrier cells. The mGM-CSF served as a stimulator to create a local inflammatory environment at the vaccination site. Importantly, any mAgs could be expressed natively on the cell adjuvant using a simple transient transfection method. We applied this cell adjuvant successfully to two potential mAgs, surface EVI2B (a type I single-pass transmembrane protein) and CXCR2 (a type IV multi-pass transmembrane GPCR). Thus, this cell adjuvant has the potential to be applied into various mAgs that are valuable but hard to obtain, such as GPCR or ion channel gate proteins. This simple and convenient method may help the production of anti-mAg functional Abs, thus accelerating Ab drug development.

Using a native conformation of mAgs to immunize animals is one key to producing functional Abs, particularly for those against multi-pass membrane proteins. Generally, expression of recombinant GPCRs by *E. coli* easily forms inclusion bodies, causing protein misfolding^16^. Antibodies raised by the misfolded mAgs are difficult to bind the native epitopes that are important for activating or blocking downstream signaling pathways^17,18^. Although linear and cyclic peptides could directly mimic the epitopes within the extracellular domain of GPCR, the conformation of these peptides is distinct from the original second and tertiary structure of mAgs^19^. Jensen and colleagues reported that ten commercial Abs that were generated by a synthetic peptide of the α_1_-adrenergic receptor failed to recognize the same receptor in mouse tissues^20^. Thus, it is important to use a native form of mAgs to immunize animals. Here, we expressed the full-length of EVI2B and CXCR2 directly on the surface of cell adjuvants. That is exactly where mAgs are originally expressed. Moreover, the lipid bilayer can stabilize the native homodimer or heterodimer structures of GPCRs that are hard to preserve as recombinant proteins or synthetic peptides^21^. Therefore, using this cell adjuvant to express mAgs could preserve their native structures on the cell membrane, which may help induce functional Abs production.

Acquisition of mAgs without extraction and purification procedures may accelerate Ab development. Most multi-pass mAgs are solubilized and isolated from the membrane by detergent. Since these mAgs are prone to misfolding, aggregation, or degradation during membrane extraction, the choice of detergent is critical and depends on the property of each mAg. With GPCRs for instance, the mild detergent n-Dodecyl-β-D-Maltoside (DDM) which was utilized human A_2A_ adenosine receptor^22^ was unable to stabilize β_2_–adrenergic receptor; hence, it was replaced by an additional detergent, maltose neopentyl glycol-3 (MNG-3)^23^. Moreover, for those mAgs enriched in the lipid raft, such as gonadotrophin-releasing hormone (GnRH) receptor or C-C chemokine receptor 5 (CCR5), lipid disrupting chemicals (nystatin or filipin) are needed^24,25^. Thus, the selection of proper detergent is largely based on rules-of-thumb to optimize the extraction and purification procedure for each mAg which may be costly and time-consuming. As a result, it is important to develop a universal platform for various mAgs. Using our strategy, EVI2B and CXCR2 were directly expressed on the cell adjuvant by transient transfection to generate mAg-expressing cell adjuvants, which were ready for immunization without complicated manipulation. The procedure of transient transfection is a commonly used method for protein expression, and the protocols for various mAgs are similar. Thus, using the cell adjuvant to express mAgs for animal immunization is a simple and convenient method, which may accelerate Ab drug development.

The important characteristics of an immunization adjuvant are to create a local pro-inflammatory environment and offer sustained Ag stimulation. Conventionally, heat-killed mycobacterial cells are used in Freund’s Complete Adjuvant (FCA) as an immune-stimulator to induce a local inflammatory response at the vaccination site^26^. It produced 1.5–2 fold higher productivity of human IgG Ab compared to those without the immune-stimulator in both rabbits and chickens^27^. On the other hand, continuous long-term Ag stimulation is essential to generate sustained high titers of antibody responses^28^. Herbert showed that ovalbumin in water-in-mineral oil emulsion, a formulation of slow released adjuvant, effectively stimulated Ab response lasting up to 544 days and induced 500 times higher Ab tilter in injected mice than the mice without adjuvant injection^29^. To mimic the conventional adjuvant, we expressed cytokines as membrane-bound forms that can induce local inflammation. The stable expression of membrane-bound cytokines also offers sustained mAg stimulation. We showed that mGM-CSF was effective in stimulating splenocyte proliferation *in vitro*. It also induced 3-fold higher anti-EVI2B Ab responses compared to the control cells. Moreover, using CXCR2 expressing mGM-CSF cell adjuvant also induced anti-CXCR2 Ab responses in mice. Thus, our cell adjuvant may follow the principles of the conventional adjuvant used in immunization procedures to induce anti-mAg Ab responses.

We successfully established a syngeneic cell adjuvant by stably expressing membrane-bound GM-CSF on BALB/3T3 cells, which could be applied for any target mAg by simple transient transfection for BALB/c mouse immunization. Then, using mAg-expressing 293 A cells for Ab characterization was able to eliminate non-specific binding. Thus, the advantages of this platform are: (1) the preservation of native mAg, (2) simple and similar processes for various mAgs, (3) stimulating local and sustained pro-inflammation at the vaccination site, and (4) the concept can also be applied to the corresponding species (e.g., RFL-6 cell to Sprague-Dawley rats, Rat2 cells to Fischer rats, RAB-9 cells for New Zealand white rabbits). Although the syngeneic cell adjuvant also induced non-specific Ab responses in the immunized mice, further studies to increase the response against the desired mAg are needed. Taken together, we believe that this platform is convenient and effective and is applicable to any mAg and may be helpful for Ab drug development.

## Supplementary information

Supplementary Information
