# Supplementary material for: Use of syngeneic cells expressing membrane-bound GM-CSF as an adjuvant to induce antibodies against native multi-pass transmembrane protein
Source: Sci Rep. 2019 Jul 9;9:9931. doi: 10.1038/s41598-019-45160-9 (PMC6616555; doi:10.1038/s41598-019-45160-9)
Supplement: Supplementary file 1 — Supplementary Information [file 41598_2019_45160_MOESM1_ESM.docx]

**Use of syngeneic cells expressing membrane-bound GM-CSF as an adjuvant to induce antibodies against native multi-pass transmembrane protein**

Chien-Chiao Huang^1, #^, Kai-Wen Cheng^1, #^, Yuan-Chin Hsieh^1^, Wen-Wei Lin^1, 2, 3, 4^, Chiu-Min Cheng^5^, Shyng-Shiou F. Yuan^6, 7, 8^, I-Ju Chen^1^, Yi-An Cheng^2^, Yun-Chi Lu^2^, Bo-Cheng Huang^9^, Yi-Ching Tung^10, *^, Tian-Lu Cheng^1, 2, 4, 9, 11, *^

^1^Center for Biomarkers and Biotech Drugs, Kaohsiung Medical University, Kaohsiung, Taiwan;

^2^Graduate Institute of Medicine, College of Medicine, Kaohsiung Medical University, Kaohsiung, Taiwan;

^3^Department of Laboratory Medicine, School of Medicine, College of Medicine, Kaohsiung Medical University, Kaohsiung, Taiwan;

^4^Department of Medical Research, Kaohsiung Medical University Hospital, Kaohsiung, Taiwan;

^5^Department and Graduate Institute of Aquaculture, National Kaohsiung University of Science and Technology, Kaohsiung, Taiwan;

^6^Translational Research Center, Kaohsiung Medical University Hospital, Kaohsiung Medical University, Kaohsiung, Taiwan;

^7^Department of Medical Research and Department of Obstetrics and Gynecology, Kaohsiung Medical University Hospital, Kaohsiung Medical University, Kaohsiung, Taiwan;

^8^School of Medicine, College of Medicine, Kaohsiung Medical University, Kaohsiung, Taiwan;

^9^Institute of Biomedical Sciences, National Sun Yat-sen University, Kaohsiung, Taiwan;

^10^Department of Public Health and Environmental Medicine, College of Medicine, Kaohsiung Medical University, Kaohsiung, Taiwan;

^11^Department of Biomedical Science and Environmental Biology, Kaohsiung Medical University, Kaohsiung, Taiwan.

# Chien-Chiao Huang and Kai-Wen Cheng contributed equally to this work.

***Correspondence:**

Dr. Yi-Ching Tung, Ph.D.

Department of Public Health and Environmental Medicine, College of Medicine, Kaohsiung Medical University, No. 100, Shih-Chuan 1st Road, Kaohsiung 807, Taiwan.

Phone: 886-7-3121101-2774, Fax: 886-7-3125339, E-mail: yctung@kmu.edu.tw

Dr. Tian-Lu Cheng, Ph.D.

Department of Biomedical and Environmental Biology, Kaohsiung Medical University, No. 100, Shih-Chuan 1st Road, Kaohsiung 807, Taiwan.

Phone: 886-7-3121101-2163-21, Fax: 886-7-3227508, E-mail: [tlcheng@kmu.edu.tw](mailto:tlcheng@kmu.edu.tw)

**
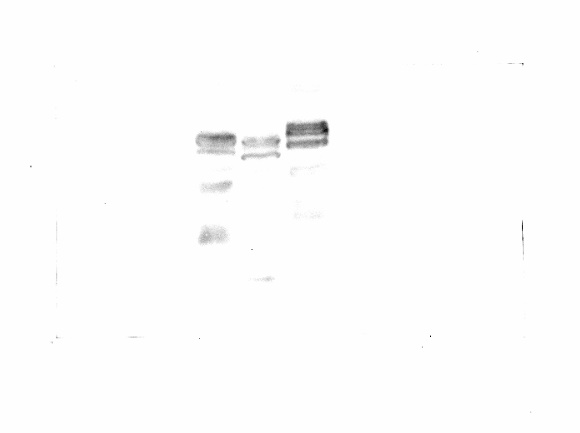
**

**
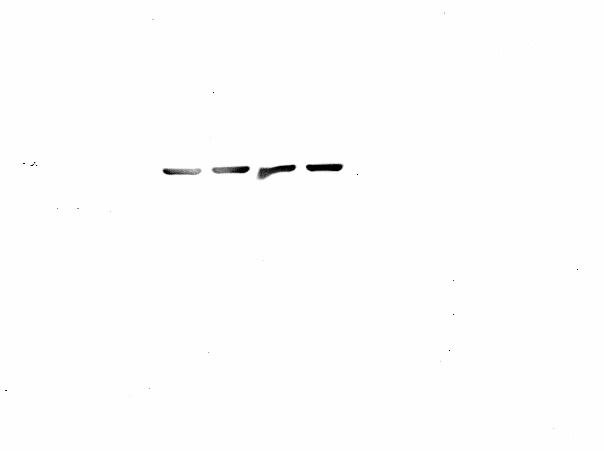
**

**Supplementary Figure 1.** Full blot images of results shown in Figure 2B.


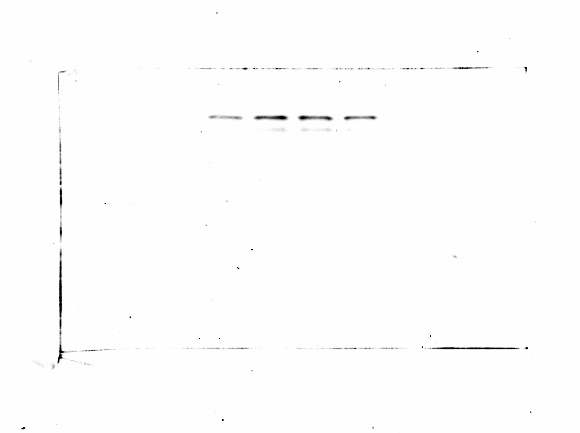


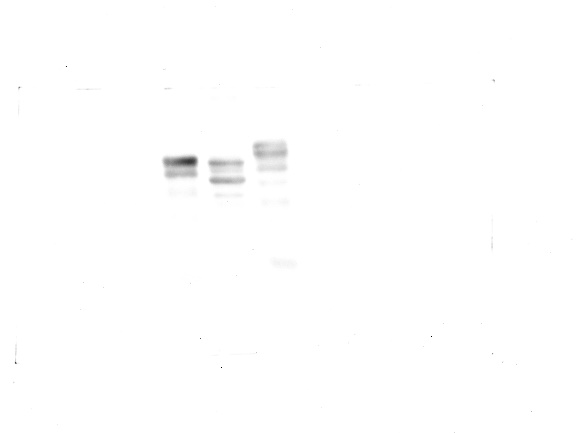


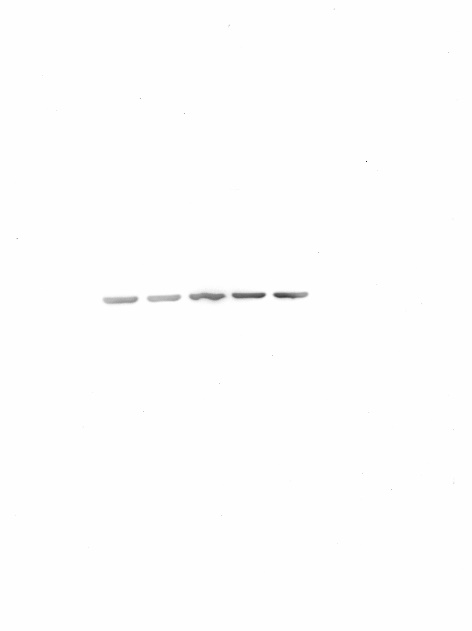


**Supplementary Figure 2.** Full blot images of results shown in Figure 4A.
